# Initial versus Staged Thyroidectomy for Differentiated Thyroid Cancer: A Retrospective Multi-Dimensional Cohort Analysis of Effectiveness and Safety

**DOI:** 10.3390/cancers16122250

**Published:** 2024-06-18

**Authors:** Eman A. Toraih, Mohammad H. Hussein, Jessan A. Jishu, Madeleine B. Landau, Ahmed Abdelmaksoud, Yaser Y. Bashumeel, Mahmoud A. AbdAlnaeem, Rithvik Vutukuri, Christine Robbie, Chelsea Matzko, Joshua Linhuber, Mohamed Shama, Salem I. Noureldine, Emad Kandil

**Affiliations:** 1 Division of Endocrine and Oncologic Surgery, Department of Surgery, School of Medicine, Tulane University, New Orleans, LA 70112, USA; mhussein1@tulane.edu (M.H.H.); aabdelmaksoud@tulane.edu (A.A.); ybashumeel@tulane.edu (Y.Y.B.); mabdalnaeem@tulane.edu (M.A.A.); mshama@tulane.edu (M.S.); 2 Genetics Unit, Histology and Cell Biology Department, Faculty of Medicine, Suez Canal University, Ismailia 41522, Egypt; 3 School of Medicine, Tulane University, New Orleans, LA 70112, USA; jjishu@tulane.edu (J.A.J.); rvutukuri@tulane.edu (R.V.); crobbie@tulane.edu (C.R.); cmatzko@tulane.edu (C.M.); jlinhuber@tulane.edu (J.L.); 4 Department of Surgery, The George Washington University School of Medicine and Health Sciences, Washington, DC 20037, USA; snoureldine@email.gwu.edu

**Keywords:** thyroid cancer, thyroidectomy, lobectomy, NSQIP, TriNetX, endocrine

## Abstract

**Simple Summary:**

The choice between total thyroidectomy and staged completion thyroidectomy for differentiated thyroid cancer remains debated. This study investigated the safety profiles and optimal timing of completion thyroidectomy by analyzing nearly 80,000 patients. The findings demonstrate that total thyroidectomy carries higher risks of temporary and permanent hypoparathyroidism compared to completion thyroidectomy. However, scheduling completion thyroidectomy within 1–6 months of the initial lobectomy can mitigate permanent complication rates. These results provide insights to guide personalized surgical decision-making for thyroid cancer patients.

**Abstract:**

The optimal surgical approach for differentiated thyroid cancer remains controversial, with debate regarding the comparative risks of upfront total thyroidectomy versus staged completion thyroidectomy following the initial lobectomy. This study aimed to assess the complication rates associated with these two strategies and identify the optimal timing for completion thyroidectomy using a multi-dimensional analysis of four cohorts: an institutional series (*n* = 148), the National Surgical Quality Improvement Program (NSQIP) database (*n* = 39,992), the TriNetX repository (*n* > 30,000), and a pooled literature review (10 studies, *n* = 6015). Institutional data revealed higher overall complication rates with total thyroidectomy (18.3%) compared to completion thyroidectomy (6.8%), primarily due to increased temporary hypocalcemia (10% vs. 0%, *p* = 0.004). The NSQIP analysis demonstrated that total thyroidectomy was associated with a 72% increased risk of transient hypocalcemia (*p* < 0.001) and a 25% increased risk of permanent hypocalcemia (*p* < 0.001). TriNetX data confirmed these findings and identified obesity and concurrent neck dissection as risk factors for complications. A meta-analysis showed that total thyroidectomy increased the rates of transient (RR = 1.63) and permanent (RR = 1.23) hypocalcemia (*p* < 0.001). Institutional and TriNetX data suggested that performing completion thyroidectomy between 1 and 6 months after the initial lobectomy minimized permanent complication rates compared to delays beyond 6 months. In conclusion, for differentiated thyroid cancer, total thyroidectomy is associated with higher risks of transient and permanent hypocalcemia compared to staged completion thyroidectomy. However, performing completion thyroidectomy within 1–6 months of the initial lobectomy may mitigate the risk of permanent complications. These findings can inform personalized surgical decision-making for patients with differentiated thyroid cancer.

## 1. Introduction

Thyroid cancer (TC) represents the most rapidly increasing cancer diagnosis in the United States, with the incidence tripling over recent decades [1]. Differentiated subtypes constitute over 90% of cases and have an excellent prognosis when appropriately treated [2]. However, debate persists regarding the optimal initial surgical management for low-to-intermediate-risk differentiated thyroid cancer (DTC) [2].

Standard therapy requires surgery, either an initial thyroid lobectomy or total thyroidectomy (TT) [3]. Although a thyroid lobectomy is recommended for low-risk DTC with small tumor size without local invasion or nodal metastases [4,5], 25–85% of resection specimens reveal worrisome features like multifocality or lymphovascular invasion that prompt completion thyroidectomy (cT) for proper staging and adjuvant therapy eligibility [6,7]. Staged cT is generally warranted for patients with lower-risk disease characteristics (tumors < 4 cm, absence of extrathyroidal extension or bulky lymphadenopathy) who have indications for completion surgery based on final pathology from the initial lobectomy. However, some of the literature suggests that cT escalates complication risks from repeat operations and contralateral lobe devascularization [8,9,10], while TT remains the preferred approach for more aggressive cancers.

Prior analyses have conflicting findings regarding the risks of TT versus cT. Some studies report lower complication rates and equivalent oncologic outcomes with cT [11,12,13]. However, others reveal increased recurrent laryngeal nerve (RLN) injuries or hypoparathyroidism without differences in recurrence rates [14,15,16,17]. Other studies found no significantly different outcomes between TT and cT, but these conclusions also require more support [12,18,19]. Defining the optimal surgical strategy and timing remains actively debated to balance risks against prognosis [8,20,21], considering factors such as costs, anesthesia exposures, and patient preferences. In addition to oncologic considerations, patient preferences play a crucial role in determining the optimal surgical approach for DTC. Patients may have varying perspectives on the trade-offs between complication risks and the burdens of undergoing a staged procedure, which should be factored into individualized decision-making.

Currently, there is no consensus on whether one-stage TT or two-stage cT thyroidectomy is associated with greater risks [13,14,17,22,23]. As more DTC cases are treated surgically [24], it is important to investigate outcomes following these procedures. We aimed to perform an extensive analysis of DTC patients undergoing TT or cT using four patient cohorts: (1) an institutional series of 148 patients; (2) a national sample of nearly 40,000 cases; (3) a multicenter database of over 30,000 cases; (4) a pooled literature review of over 7000 patients. By triangulating evidence across these diverse datasets, we sought to provide a rigorous and generalizable comparison of complication rates between surgical approaches. An ancillary objective was examining cT timing thresholds. These real-world data assessing comparative safety and effectiveness can facilitate personalized recommendations through risk–benefit profiling, enabling surgeons and patients to select an approach that aligns with individualized needs and values when oncologically appropriate.

## 2. Materials and Methods

### 2.1. Institutional Cohorts

#### 2.1.1. Study Design

We conducted a retrospective cohort study following Institutional Review Board approval (#2023-452). The study period spanned 2010–2021, with an average follow-up period of 33.6 months (ranging from 1 to 115 months). Patients undergoing TT or cT for DTC at our institution were included. TT was defined as single-stage total resection, while cT consisted of removing the remaining lobe in those with a prior contralateral lobectomy.

#### 2.1.2. Study Population

Patients were included if they were undergoing either TT or cT for thyroid cancer without prior neck surgery or radioactive iodine therapy. Exclusion criteria were loss to follow-up or incomplete data.

#### 2.1.3. Study Variables

The parameters collected encompassed demographics, comorbidities, pathology details, genomic mutation status, American Thyroid Association recurrence risk stratification, and treatment modalities. Postoperative calcium and PTH levels were collected for patients who developed biochemical hypocalcemia (defined as total serum calcium < 8.5 mg/dL or ionized calcium < 1.0 mmol/L) within 30 days of surgery. The lowest recorded calcium and PTH values during this period were used for analysis.

#### 2.1.4. Study Outcomes

The surgical complications assessed were hematoma, seroma, recurrent laryngeal nerve (RLN) injury, and hypocalcemia (calcium < 8.5 mg/dL or 2.12 mmol/L). Complications were categorized as temporary (<6 months duration) or permanent. Oncologic outcomes included recurrence events and disease-free and overall survival. Additional subgroup analysis compared 40 TT to 53 cT patients with tumor size ≤ 2 cm for complication rates.

### 2.2. NSQIP Database

#### 2.2.1. Data Source

Data were obtained from the NSQIP database, an outcomes-based program established by the American College of Surgeons. The database collects information on patient demographics, preoperative risk factors, intraoperative variables, and 30-day postoperative outcomes [25]. It allows participating hospitals to benchmark their surgical outcomes, identify areas for improvement, and implement targeted strategies to enhance patient care.

#### 2.2.2. Study Population

The study population included thyroid cancer patients who underwent thyroid surgery from 2007 to 2020. The inclusion criteria were based on CPT procedure codes (Supplementary Table S1). Patients with incomplete records, previous neck surgery, radiation therapy, a prior history of thyroid cancer, or non-thyroid malignancies were excluded. Those who had a postoperative diagnosis of congenital thyroid abnormalities, unspecified thyroid disease, secondary malignancies, or parathyroid disorders were also excluded.

#### 2.2.3. Study Variables

The variables examined included patient demographics and comorbidities such as diabetes, hypertension, congestive heart failure, etc. The American Society of Anesthesiologists (ASA) classification was used to assess a patient’s physical health prior to anesthesia and surgery. Surgical variables included surgeon specialty, surgery duration, and transfusion requirements.

#### 2.2.4. Study Outcomes

This study focused on comparing operative procedures and complication rates among cohorts. It analyzed various parameters, including operative time, transfusion rates, return to the operating room, length of stay, and various complications. Complications of interest included bleeding, superficial incisional infection, RLN injury, postoperative hypocalcemia, sepsis, septic shock, deep vein thrombosis/pulmonary embolism, and pneumonia.

#### 2.2.5. Subgroup Analysis

An additional analysis was conducted to assess postoperative hypocalcemia risk stratified by neck dissection (ND) status among TT groups compared to the cT cohort. Thirty-day hypocalcemia rates were compared via Chi-square analysis. Relative risk (RR) and 95% confidence intervals were calculated using cT as the reference group.

### 2.3. TriNetX Database

#### 2.3.1. Data Source

We retrospectively analyzed de-identified electronic health records for over 135 million patients from 112 healthcare organizations across 16 countries, including the United States, via the TriNetX research platform. This analysis followed the Strengthening the Reporting of Observational Studies in Epidemiology STROBE (guidelines) for reporting observational studies.

#### 2.3.2. Study Population

The study population included thyroid cancer patients who underwent thyroidectomy, identified using CPT procedure codes (Supplementary Table S1). Patients with incomplete records were excluded from the analysis.

#### 2.3.3. Study Variables

The details extracted encompassed demographics (age, sex, race, ethnicity, body mass index), comorbidities (obesity, diabetes, hypertension, cerebrovascular/respiratory/kidney disease), cancer characteristics (pathological tumor size, nodal stage, distant metastasis), and treatments (TT, cT, central/lateral neck dissection).

#### 2.3.4. Study Outcomes

The primary outcomes analyzed encompassed post-thyroidectomy complications, specifically temporary and permanent hypocalcemia and RLN injury. Hypocalcemia was defined as calcium < 8.5 mg/dL. The duration was defined as transient (lasting < 6 months) versus permanent (>6 months) based on the persistence of sequela or the need for ongoing supplementation. The prevalence and RR of complications were compared between TT and cT groups.

#### 2.3.5. Subgroup Analysis

Analyses stratified patients by (1) obesity status (non-obese vs. obese), (2) neck dissection extent (TT with ND vs. cT alone; limited vs. radical ND), and (3) completion thyroidectomy timing from initial surgery (1–15 days; 16–30 days; 1–3 months; 3–6 months; >6 months).

### 2.4. Literature Screening

#### 2.4.1. Systemic Search

A systematic search was conducted using PubMed, ScienceDirect, and Google Scholar databases to identify studies comparing TT to cT for DTC. The search strategy incorporated terms related to “differentiated thyroid cancer”, “total thyroidectomy”, “completion thyroidectomy”, and associated Medical Subject Headings terminology. Studies published in English through January 2024 were considered.

#### 2.4.2. Eligibility Criteria

The inclusion criteria consisted of comparative studies analyzing complication rates between TT and cT for differentiated thyroid carcinoma. Eligible study designs included randomized trials, cohort studies, and case–control studies. Case reports, reviews, abstracts, and non-English studies were excluded.

#### 2.4.3. Data Extraction

Four reviewers independently screened studies for eligibility and extracted data using predefined criteria. The extracted information included year, country, study design, sample size, population characteristics, and rates of complications, including nerve injuries and hypocalcemia. Disagreements in extraction were resolved by consensus.

### 2.5. Statistical Analysis

The statistical analysis was performed using R Studio version 2022.07.1 Build 554 (R Foundation for Statistical Computing, Vienna, Austria). Descriptive statistics included frequencies and percentages for categorical means and standard deviations or medians and interquartile ranges for continuous variables. Pearson’s Chi-square or Fisher’s exact tests were used for categorical variables, and Mann–Whitney U or Student’s *t*-tests for continuous variables. Logistic regression models were used to identify predictors of surgical complications or disease recurrence, reporting odds ratios and 95% confidence intervals (95%CI). Time-to-event analysis was conducted using Kaplan–Meier curves to estimate survival probabilities over time, with the log-rank test evaluating differences between groups. Univariate Cox proportional hazards regression quantified hazard ratios for factors impacting disease-free and overall survival. Significant variables were further assessed in multivariate Cox models to determine independent prognostic variables. All statistical tests were two-tailed, and a significance level of *p* < 0.05 was used to determine statistical significance. The DerSimonian–Laird approach with the Mantel–Haenszel method was used for pairwise comparisons to calculate pooled RR and 95%CI. The “Metafor” R package was used.

## 3. Results

### 3.1. Institutional Cohort Analysis

#### 3.1.1. Characteristics of the Study Population

The study included 148 DTC patients, comprising 137 papillary thyroid carcinomas (PTCs) and 11 follicular thyroid carcinomas (FTCs). Of these, 60 underwent TT, and 88 underwent cT. No patients had distant metastasis at presentation. There were no significant demographic or pathological differences between the TT and cT groups in terms of mean age (51.7 ± 14.4 years for TT vs. 52.1 ± 15.2 years for cT, *p* = 0.70), gender (81.7% female for TT vs. 81.8% for cT, *p* = 0.98), racial background (31.7% African American for TT vs. 38.6% for cT, *p* = 0.48), body mass index (30.6 ± 7.6 kg/m^2^ for TT vs. 32.2 ± 8.3 kg/m^2^ for cT, *p* = 0.24), tumor size (1.2 cm, IQR = 0.4–2.1 for TT vs. 1.56 cm, IQR = 0.9–2.4 for cT, *p* = 0.14), or the rate of intermediate/high-risk cancers per ATA criteria (29.3% for TT vs. 26.2% for cT, *p* = 0.88), as shown in Table 1.

Among patients who developed biochemical hypocalcemia, the mean postoperative nadir total serum calcium level was 7.8 ± 0.4 mg/dL in the total thyroidectomy group and 8.1 ± 0.3 mg/dL in the completion thyroidectomy group (*p* = 0.09). The mean postoperative nadir PTH level was 12.6 ± 8.2 pg/mL in the total thyroidectomy group and 18.4 ± 11.5 pg/mL in the completion thyroidectomy group (*p* = 0.14).

Among the 148 patients undergoing thyroidectomy at our center, there were no significant differences in the mean number of parathyroid glands inadvertently excised (0.3 ± 0.6 for total thyroidectomy vs. 0.2 ± 0.5 for completion thyroidectomy, *p* = 0.28) between the two surgical groups.

#### 3.1.2. Postoperative Complications and Disease Outcomes

Complications occurred more frequently in the TT group (18.3%) compared to the cT group (6.8%) (*p* = 0.038). This was driven by increased temporary hypocalcemia among TT patients (10% for TT vs. 0% for cT, *p* = 0.004). No permanent RLN injuries or permanent hypocalcemia events occurred. No mortality events occurred during follow-up. Only two patients (1.4%) suffered disease recurrence during a median follow-up of 24.4 months (IQR 5.0–55.3 months), with no significant differences in recurrence rates (*p* = 0.16), disease-free survival durations (*p* = 0.19), or overall survival times (*p* = 0.16) between surgical groups, as outlined in Table 1. On multivariate analysis, no factors independently predicted complications among all patients, as shown in Supplementary Table S2.

#### 3.1.3. Subgroup Analysis for Small Tumors

Among 93 patients with small tumors, 40 underwent TT, and 53 underwent cT. The complication rate remained higher for TT patients (22.5%) compared to cT patients (7.5%), although this was not significant (*p* = 0.07). Temporary hypocalcemia occurred more frequently with TT (12.5%) versus cT (0%) for tumors < 2 cm in size (*p* = 0.013). No permanent RLN injuries, permanent hypocalcemia, wound complications, or hematomas/seromas were reported for either surgical group (Supplementary Table S3).

### 3.2. NSQIP Data Analysis

#### 3.2.1. Cohort Selection Process from the NSQIP Dataset

The initial NSQIP cohort undergoing thyroid surgery consisted of 201,015 patients. Cases with undefined surgical procedures were first excluded (*n* = 6122), followed by the exclusion of partial thyroidectomies (*n* = 73,980) or missing demographics (*n* = 117). This left 112,737 patients undergoing one-stage total thyroidectomies and 8176 undergoing completion thyroidectomies. To focus the analysis on thyroid cancer treatment, non-malignant conditions and concomitant thyroid disorders were additional exclusion criteria (*n* = 81,599), yielding a final cohort of 39,314 patients. Among these, 35,753 (90.9) had upfront total thyroidectomies, while 3561 (9.1%) had completion procedures after a prior partial thyroidectomy.

#### 3.2.2. Patient Characteristics

As shown in Table 2, the mean age of thyroidectomy patients was 50.5 ± 15.3 years, with no difference between groups (*p* = 0.13). Overall, 25.4% were male, again similar between TT and cT (*p* = 0.19). In terms of race, most patients were White (84.6%), although Black patients made up a greater proportion of cT cases (10.5%) versus TT cases (6.9%) (*p* < 0.001). The mean BMI was 30.2 ± 7.5 kg/m^2^ and was slightly higher for cT patients (30.5 ± 7.7 kg/m^2^) than TT patients (30.1 ± 7.4 kg/m^2^) (*p* < 0.001).

#### 3.2.3. Outcomes Analysis

As depicted in Table 2, most operations were performed electively by general surgeons, with shorter operative times for cT (median 89 min) than TT procedures (median 129 min) (*p* < 0.001). Of those who underwent TT, 40.4% (N = 14,700) underwent concomitant ND. Local wound complications, septicemia, and venous thromboembolic events were uncommon for both TT and cT groups (≤0.3%). Notable complications included more temporary hypocalcemia with TT (0.66%) versus cT (0.28%) (*p* = 0.007). Pneumonia and unplanned re-intubations occurred slightly more often for TT patients (*p* < 0.05). The median length of stay was 1 day for 83.6% of patients. While few patients remained hospitalized for >30 days (0.13%), readmissions were required for 2.5%, more commonly after TT (2.6%) than cT (1.6%) (*p* < 0.001). Thirty-day mortality was very low overall (0.1%), without a difference between surgical groups (*p* = 0.77).

#### 3.2.4. Subgroup Analysis Based on Neck Dissection

Among 39,314 thyroidectomy patients, 14,418 had TT with ND (12,548 central and 1870 lateral lymphadenectomies). TT patients with (RR = 2.44, 95%CI = 1.27–4.68) and without neck dissection (RR = 2.27, 95%CI = 1.19–4.31) had over twice the risk of postoperative hypocalcemia compared to those with cT, with central ND conferring the highest risk (RR = 2.52, 95%CI = 1.31–4.85). Interestingly, lateral ND does not significantly increase complications over central ND alone (RR = 0.75, 95%CI = 0.39–1.44) (Table 3).

#### 3.2.5. Hypocalcemia Predictor Analysis

A univariate logistic regression analysis was conducted to identify putative predictor risk factors of 30-day postoperative hypocalcemia (Supplementary Figure S1). Multiple demographic and clinical characteristics emerged as significant factors, including age ≥ 55 years, which was protective against hypocalcemia (OR 0.56, 95% CI 0.42–0.75, *p* < 0.001) compared to younger ages. Male gender also decreased the risk (OR = 0.39, 95%CI = 0.26–0.58, *p* < 0.001) relative to women. In contrast, obesity increased hypocalcemia odds by nearly 2-fold (OR = 1.90, 95%CI = 1.47–2.45, *p* < 0.001). Importantly, cT was associated with a 57% reduction in hypocalcemia risk compared to TT (OR = 0.43, 95%CI = 0.23–0.80, *p* = 0.008).

Upon multivariate adjustment in Supplementary Table S4, age, gender, obesity, and type of thyroidectomy remained independent predictors. The completion procedure continued to protect against hypocalcemia (OR = 0.42, 95%CI = 0.22–0.79, *p* = 0.007) versus TT.

#### 3.2.6. Predictors of Overall Mortality

Supplementary Figure S2 displays the results of the Cox regression analysis for 30-day mortality. On multivariate evaluation, diabetes mellitus emerged as the only comorbidity that increased mortality risk by over 2-fold (HR = 2.24, 95%CI = 1.06–4.72, *p* = 0.034). However, the central comparison by procedure type showed no distinction, with cT conferring similar 30-day mortality risk versus TT (unadjusted HR = 0.99, 95%CI = 0.29–3.35, *p* = 0.99). This suggests that the choice of surgical technique does not independently impact early perioperative death rates.

### 3.3. TriNetX Data Analysis

#### 3.3.1. Cohort Selection Process from the TriNetX Dataset

The TriNetX collaborative health record network contains data on over 135 million patients globally. Within this database, 244,580 patients had a diagnosis of thyroid cancer. Of those with thyroid cancer, 31,707 (86.3%) were undergoing TT, and 5039 (13.7%) were undergoing cT.

#### 3.3.2. Patient Characteristics

Supplementary Table S5 outlines the demographic and clinical features of 36,746 thyroid cancer patients undergoing single-stage TT or cT between 2000 and 2022. Statistically significant demographic differences existed between groups. cT patients were slightly older (mean 57 ± 16 years) versus TT (56 ± 17 years) with more females (71% vs. 72%). cT had a higher representation of Black (9% vs. 6%) and Hispanic (9% vs. 11%) individuals. Comorbid conditions were common, including obesity (21–22%), diabetes (21%), chronic respiratory disease (24%), hypertension (47–48%), and kidney disease (8%). Pathologically, cT patients showed modestly increased rates of lymph node metastasis (18% vs. 14%) and fewer distant metastases (2.0% vs. 3.3%).

#### 3.3.3. Risk of Postoperative Complications

The RR shows a 72% increased risk of transient hypocalcemia (95%CI = 1.62–1.81, *p* < 0.001) and a 25% increased risk of permanent hypocalcemia (95%CI = 1.15–1.36, *p* < 0.001) for patients who underwent TT versus cT. The RRs for transient and permanent RLN injury with TT versus cT were 0.93 (95%CI = 0.82–1.05, *p* = 0.22) and 0.91 (95%CI = 0.76–1.09, *p* = 0.32), respectively, as shown in Table 4.

#### 3.3.4. Subgroup Analysis

When stratified by obesity status, the risk of postoperative complications remained higher in TT versus cT patients. Among non-obese patients, TT was associated with a significantly increased risk of transient hypocalcemia (RR = 1.68; *p* < 0.001), permanent hypocalcemia (RR = 1.23; *p* = 0.005), and permanent RLN injury (RR 1.13; *p* = 0.018) compared to cT. Similarly, TT patients with obesity had a higher risk of transient hypocalcemia (RR = 1.85; *p* < 0.001) and permanent hypocalcemia (RR = 1.48; *p* < 0.001) versus the cT group (Table 4).

#### 3.3.5. Impact of Neck Dissection

When TT was accompanied by ND, the risks of postoperative complications were higher compared to cT alone. TT with ND had an increased risk of transient hypocalcemia (RR = 1.97; *p* < 0.001), permanent hypocalcemia (RR = 1.42; *p* < 0.001), transient RLN injury (RR = 1.28; *p* = 0.002), and permanent RLN injury (RR 1.24; *p* = 0.022) versus cT. Additionally, radical ND was associated with more complications than limited ND among patients undergoing TT. Radical ND had higher risks of transient hypocalcemia (RR = 1.41; *p* < 0.001), transient RLN injury (RR = 1.66; *p* < 0.001), and permanent RLN injury (RR = 1.95; *p* < 0.001) compared to limited ND. There was no difference in permanent hypocalcemia rates based on the extent of ND (RR = 1.14; *p* = 0.06) (Table 5).

#### 3.3.6. Effect of Latency Period on Complication Rates

Our analysis of 509 cT patients with a known latency period indicates 1–6 months as the optimal timing post-initial surgery to minimize permanent complications. The shortest interval of 1–15 days had the highest transient hypocalcemia rate at 37.5%, though permanent complications were comparable to cT surgery after 6 months. In contrast, permanent complication rates dropped by nearly 50% when cT occurred at 1–3 or 3–6 months compared to after 6 months. Specifically, permanent hypocalcemia rates fell to 18.0% (1–3 months) and 15.0% (3–6 months) versus 31.8% with cT after 6 months (RR = 0.57 and 0.47, respectively, both *p* < 0.01). Similarly, permanent RLN injury rates decreased from 15.3% (after 6 months) to 6.0% (1–3 months) and 5.0% (3–6 months), with RR = 0.39 (*p* = 0.012) and 0.33 (*p* = 0.018) (Table 6).

### 3.4. Literature Review

#### 3.4.1. Risk of Complications

**Figure 1:**
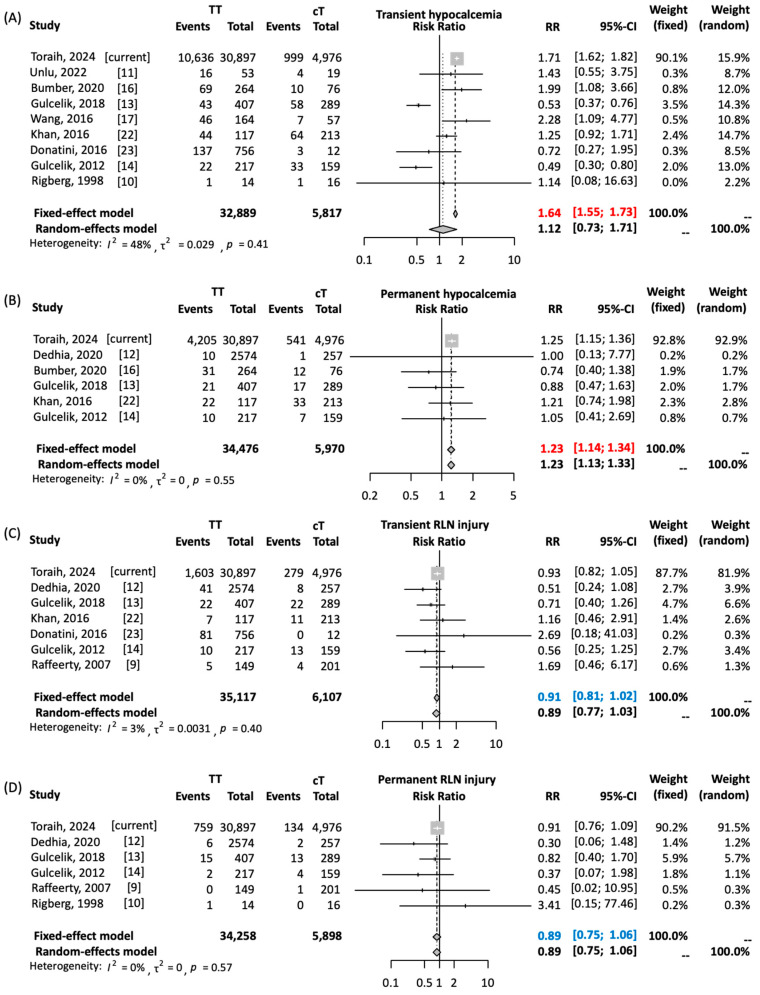
Risk of postoperative complication rates in total versus completion thyroidectomy in patients with well-differentiated thyroid cancer. (**A**) Risk of transient hypocalcemia. (**B**) Risk of permanent hypocalcemia. (**C**) Risk of transient recurrent laryngeal nerve (RLN) injury. (**D**) Risk of permanent RLN injury. Toraih’s 2024 study includes the current TriNetX analysis. Pairwise comparison was performed using Mantel–Haenszel method with DerSimonian–Laird estimator. Results are reported as relative risk (RR) and 95% confidence interval (CI). Due to absence of heterogeneity, results of fixed-effects model are reported. Red values indicate significant risk. Blue values indicate non-significant pooled estimates [9,10,11,12,13,14,16,17,22,23].

A meta-analysis of 10 cancer studies (1998–2022) comprised 4715 TT and 1299 cT patients [9,10,11,12,13,14,16,17,22,23]. We found a significantly higher risk of transient hypocalcemia with TT versus cT (RR = 1.638, 95%CI = 1.551–1.730; *p* < 0.001). TT was also associated with a small but significantly increased risk of permanent hypocalcemia (RR = 1.232, 95%CI = 1.136–1.336; *p* < 0.001). No significant differences were observed for RLN injury risks (transient RR = 0.91, *p* = 0.09; permanent RR = 0.89, *p* = 0.18), as shown in Figure 1.

#### 3.4.2. Impact of Timing of Completion Thyroidectomy

The meta-analysis found no significant differences in complication rates between early (<3 months) and delayed (≥3 months) cT (Supplementary Figure S3). The risk was not statistically significant for transient hypocalcemia (RR = 1.31, 95%CI = 0.65–2.62), permanent hypocalcemia (RR = 1.0, 95%CI = 0.98–1.03), transient RLN injury (RR = 1.67, 95%CI = 0.59–4.72), or permanent nerve injury (RR = 1.93, 95%CI = 0.31–12.2) when comparing early versus delayed cT.

## 4. Discussion

This multi-dimensional analysis of nearly 80,000 thyroidectomy patients provides valuable insights regarding safety profiles and optimal timing for completion thyroidectomy in DTC management. We demonstrated increased risks of transient and permanent hypocalcemia across cohorts, without a distinction in recurrent laryngeal nerve injury rates.

Our institutional data revealed an overall higher complication rate with total thyroidectomy, driven by increased temporary hypocalcemia. This likely relates to the more extensive bilateral dissection and parathyroid gland manipulation required for total thyroidectomy procedures. However, no permanent nerve injuries, permanent hypoparathyroidism, wound complications, hematomas/seromas, or mortality events occurred, although longer-term follow-up was limited. Further investigations with prolonged monitoring are warranted to clarify potential differences in permanent complication rates between procedures.

An examination of nearly 40,000 NSQIP cases echoed these institutional findings. TT conferred double the risk of postoperative hypocalcemia compared to cT, and this was exacerbated by central neck dissection. This reinforces that more extensive dissection escalates injury to vascularized parathyroid glands. Careful postoperative calcium monitoring and judicious supplementation are imperative, especially after comprehensive central compartment surgery.

This aligns with the TriNetX analysis of 30,000 patients demonstrating a 25–72% elevated hypocalcemia risk after TT relative to staged cT, compounded by obesity and radical lymphadenectomy. These real-world data verify that completion procedures mitigate complications as additional risk factors accumulate.

However, it is important to acknowledge that staged surgery is associated with higher costs, increased anesthesia risks, longer cumulative hospital stays, and extended total operative times compared to single-stage procedures. While our analysis focuses on the decreased hypocalcemia rates with staging, surgeons and patients must weigh the full range of trade-offs when considering staged surgery, including resource utilization, hospital stays, and total operative times. Cost-analysis studies evaluating the trade-offs between the two approaches in light of their complication profiles would help guide optimal surgical decision-making.

Incorporating patient preferences is also crucial when comparing surgical strategies. While staged procedures may offer advantages in terms of reducing certain complication rates, they also involve additional surgery, anesthesia, and recovery time, which may be undesirable for some patients. Thorough patient counseling and shared decision-making are essential in weighing the risks and benefits of staged versus single-stage procedures. Surgeons should provide clear information on the potential complications, recovery process, and long-term outcomes associated with each approach. Patient preferences may vary based on individual values, lifestyle factors, and personal experiences. Some patients may prioritize minimizing the risk of complications like transient hypocalcemia, while others may prefer to avoid multiple surgeries. The decision between a single-stage or staged procedure should be made collaboratively between the surgeon and patient, taking into account the patient’s specific cancer characteristics, overall health status, and personal preferences. Future research should incorporate patient-reported outcomes and quality-of-life measures to better understand the impact of different surgical approaches on patient experiences and satisfaction.

Similarly, a pooled meta-analysis of over 6000 published cases across a wide geographic region further supports the increased transient (64%) and permanent (23%) hypocalcemia seen with TT. However, there was no significant difference between the two surgical procedures regarding nerve injury rates. The lack of permanent laryngeal nerve injury in each treatment group can possibly be attributed to the experience and surgical volume of the surgeon as well as the use of postoperative laryngoscopy rather than the type of surgery itself [26,27].

The optimal timing for cT remains controversial. Some studies have found no difference in complication rates when cT is performed between 10 and 90 days versus after 90 days from the initial thyroid surgery. However, other evidence suggests that delayed completion thyroidectomy lowers complication risks [8,20,21]. Based on the TriNetX analysis, we determined 1–6 months following lobectomy as the “optimal window” with the lowest complication rates compared to later or earlier intervals. Our meta-analysis demonstrated no differences in complication rates based on a 3-month threshold for early versus delayed completion. This indicates a potential benefit in staging operations during the 1–6-month window to mitigate long-term risks of hypocalcemia and recurrent laryngeal nerve injury. The precise mechanism is unclear but may relate to avoiding extremes of acute inflammation with very early reoperation versus advanced scarring and anatomical distortions with later completion procedures.

This study has certain limitations. All datasets, apart from our single-center cohort, lacked granularity on surgical techniques, the extent of resection, and postoperative supplementation, which could modify complication rates. In addition, the meta-analysis was relatively underpowered to detect differences between granular timing thresholds. However, the rigorous compilation of evidence across institutional, multicenter, population-based, and literature-derived datasets lends reliability with complementary results.

Moving forward, prospective multicenter registries can refine personalized decision algorithms through better patient and procedure characterizations. Cost analyses should also evaluate staged completion approaches in relation to hospital resources. Ultimately, delineating the precise trade-offs between surgical risks and oncologic outcomes can advance individualized and value-based thyroid cancer care.

## 5. Conclusions

This aggregate analysis demonstrates increased transient and permanent hypocalcemia risks with TT versus cT for DTC. The real-world data also identified an opportune 1–6-month completion window that positively impacts permanent postoperative sequelae. However, staged procedures also carry trade-offs in terms of increased costs, anesthesia exposures, cumulative hospital stays, and total operative times that must be weighed against their complication benefits. Factoring in patient preferences through shared decision-making is essential to tailor surgical strategies to individual needs and values. Randomized controlled data could help further clarify optimal completion thyroidectomy timing thresholds that best balance oncologic outcomes with morbidity. These multi-dimensional insights carry useful implications for surgical decision-making through individualized risk–benefit profiling. Assessing these nuances can guide staged and timely completion thyroidectomy approaches to optimize care for DTC.

## Figures and Tables

**Table 1:** Characteristics and outcomes of thyroidectomy patients at our institution.

Characteristics	Total (N = 148)	TT (N = 60)	cT (N = 88)	*p*-Value
**Demographic**				
Age, years	51.9 ± 14.9	51.7 ± 14.4	52.1 ± 15.2	0.70
Female sex	121 (81.8)	49 (81.7)	72 (81.8)	0.98
African American	53 (35.8)	19 (31.7)	34 (38.6)	0.48
BMI, Kg/m^2^	31.5 ± 8.0	30.6 ± 7.6	32.2 ± 8.3	0.24
**Comorbidities**				
Hashimoto thyroiditis	23 (15.5)	11 (18.3)	12 (13.6)	0.49
Family history of thyroid cancer	21 (14.2)	12 (20)	9 (10.2)	0.15
Prior radiation exposure	7 (4.7)	3 (5)	4 (4.5)	0.89
**Pathology**				
Max diameter, cm	1.5 (0.55–2.4)	1.2 (0.4–2.1)	1.56 (0.9–2.4)	0.14
≤2 cm	93 (66.4)	40 (71.4)	53 (63.1)	0.36
>2 cm	47 (33.6)	16 (28.6)	31 (36.9)	
T stage				
T1/2 stage	133 (89.9)	51 (85)	82 (93.2)	0.16
T3/4 stage	15 (10.1)	9 (15.0)	6 (6.8)	
N stage				
N0 stage	138 (93.2)	55 (91.7)	83 (94.3)	0.52
N1 stage	10 (6.8)	5 (8.3)	5 (5.7)	
Central LNM	9 (6.1)	4 (6.7)	5 (5.7)	0.81
Multifocal	64 (43.2)	27 (45)	37 (42)	0.74
Bilateral lesions	39 (26.4)	18 (30)	21 (23.9)	0.45
Capsular invasion	29 (19.6)	8 (13.3)	21 (23.9)	0.14
Extrathyroidal extension	9 (6.1)	4 (6.7)	5 (5.7)	0.81
Extranodal extension	2 (1.4)	1 (1.7)	1 (1.1)	0.78
**Genomic screening**				
BRAF mutation	43 (29.1)	17 (28.3)	26 (29.5)	0.87
TERT mutation	1 (0.7)	1 (1.7)	0 (0)	0.41
RAS mutation	12 (8.1)	2 (3.3)	10 (11.4)	0.12
**ATA risk stratification**				
Low risk	107 (72.3)	42 (70)	65 (73.9)	0.88
Intermediate risk	32 (21.6)	14 (23.3)	18 (20.5)	
High risk	9 (6.1)	4 (6.7)	5 (5.7)	
**Laboratory data**				
Serum calcium, mg/dL	7.9 ± 0.4	7.8 ± 0.4	8.1 ± 0.3	0.09
PTH level, pg/mL	15.2 ± 10.1	12.6 ± 8.2	18.4 ± 11.5	0.14
**Treatment**				
Postoperative RAI	39 (26.4)	18 (30)	21 (23.9)	0.45
**Complications**				
Any complication	17 (11.5)	11 (18.3)	6 (6.8)	**0.038**
Hematoma/seroma	0 (0)	0 (0)	0 (0)	NA
Wound complications	1 (0.7)	1 (1.7)	0 (0)	0.41
Temporary RLN dysfunction	12 (8.1)	6 (10)	6 (6.8)	0.55
Permanent RLN dysfunction	0 (0)	0 (0)	0 (0)	NA
Temporary hypocalcemia	6 (4.1)	6 (10)	0 (0)	**0.004**
Permanent hypocalcemia	0 (0)	0 (0)	0 (0)	NA
**Oncological outcomes**				
Recurrence	2 (1.4)	2 (3.3)	0 (0)	0.16
Disease-free survival, months	24.4 (5.0–55.3)	34.8 (5.2–58.9)	20.5 (4.1–53.1)	0.19
Overall survival time, months	25.2 (5.03–55.6)	35.1 (5.1–64.4)	20.6 (4.2–53.0)	0.16

Data are reported as count (percentage), mean ± standard deviation, or median (interquartile range). Two-sided Chi-square, Student’s t, or Mann–Whitney U tests were used. Bold *p*-values indicate statistical significance < 0.05. TT: total thyroidectomy; cT: completion thyroidectomy; BMI: body mass index; FTC: follicular thyroid cancer; PTC: papillary thyroid cancer; PTMC: papillary thyroid microcarcinoma; LNM: lymph node metastasis; RAS: includes NRAS, HRAS, OR KRAS; RAI: radioactive iodine ablation; RLN: recurrent laryngeal nerve injury.

**Table 2:** Characteristics and outcomes of thyroidectomy patients in NSQIP dataset.

Characteristics	Overall	TT	cT	*p*-Value
**Number**	39,314	35,753	3561	
**Demographics**				
Age, years	50.5 ± 15.3	50.4 ± 15.3	50.8 ± 15.2	0.13
<30	4360 (11.09%)	3974 (11.12%)	386 (10.84%)	0.23
30–49	15,075 (38.35%)	13,759 (38.48%)	1316 (36.96%)	
50–69	15,860 (40.34%)	14,381 (40.22%)	1479 (41.53%)	
≥70	4019 (10.22%)	3639 (10.18%)	380 (10.67%)	
Male sex	9995 (25.42%)	9057 (25.33%)	938 (26.34%)	0.19
Race				
White	33,249 (84.57%)	30,309 (84.77%)	2940 (82.56%)	**<0.001**
Black	2843 (7.23%)	2469 (6.91%)	374 (10.5%)	
Asian	3048 (7.75%)	2818 (7.88%)	230 (6.46%)	
AI/AN	174 (0.44%)	157 (0.44%)	17 (0.48%)	
Hispanic/Latino	3372 (8.58%)	3101 (8.67%)	271 (7.61%)	**0.031**
BMI, Kg/m^2^	30.2 ± 7.5	30.1 ± 7.4	30.5 ± 7.7	**<0.001**
**Comorbidities**				
Obesity (BMI > 30 Kg/m^2^)	17,209 (43.77%)	15,546 (43.48%)	1663 (46.7%)	**<0.001**
Smoking within one year	4431 (11.27%)	4083 (11.42%)	348 (9.77%)	**0.003**
Diabetes	5094 (12.96%)	4626 (12.94%)	468 (13.14%)	0.37
Hypertension requiring medication	14,213 (36.15%)	12,864 (35.98%)	1349 (37.88%)	**0.024**
Severe COPD	702 (1.79%)	647 (1.81%)	55 (1.54%)	0.29
Current pneumonia	8 (0.02%)	8 (0.02%)	0 (0%)	0.37
Congestive heart failure within 30 d	78 (0.2%)	75 (0.21%)	3 (0.08%)	0.16
Transient ischemic attack	113 (0.29%)	101 (0.28%)	12 (0.34%)	0.51
CVA/stroke with deficit	76 (0.19%)	71 (0.2%)	5 (0.14%)	0.55
CVA/stroke without deficit	66 (0.17%)	61 (0.17%)	5 (0.14%)	0.83
Acute renal failure	17 (0.04%)	14 (0.04%)	3 (0.08%)	0.19
On dialysis	141 (0.36%)	132 (0.37%)	9 (0.25%)	0.31
Bleeding disorders	448 (1.14%)	418 (1.17%)	30 (0.84%)	0.09
Immunosuppressive or Steroid	893 (2.27%)	817 (2.29%)	76 (2.13%)	0.59
Weight loss in last 6 months	184 (0.47%)	179 (0.5%)	5 (0.14%)	**0.003**
**ASA classification**				
ASA Class I	2360 (6%)	2199 (6.15%)	161 (4.52%)	**<0.001**
ASA Class II	24,018 (61.09%)	21,878 (61.19%)	2140 (60.1%)	
ASA Class III	12,429 (31.61%)	11,205 (31.34%)	1224 (34.37%)	
ASA Class IV	463 (1.18%)	432 (1.21%)	31 (0.87%)	
ASA Class V	1 (0%)	1 (0%)	0 (0%)	
**Pathological extension**				
Disseminated cancer	890 (2.26%)	822 (2.3%)	68 (1.91%)	0.14
**Surgical data**				
Elective surgery	31,788 (80.86%)	28,880 (80.78%)	31,788 (80.86%)	0.20
Surgery specialty				
General surgery	27,459 (69.85%)	25,173 (70.41%)	2286 (64.2%)	**<0.001**
Otolaryngology	11,855 (30.15%)	10,580 (29.59%)	1275 (35.8%)	
Operative procedure				
Operative time, min	125 (90–174)	129 (94–178)	89 (65.5–121)	**<0.001**
Transfusions	80 (0.2%)	79 (0.22%)	1 (0.03%)	**0.010**
Return to OR	540 (1.37%)	511 (1.43%)	29 (0.81%)	**0.003**
**Complications**				
Local complications				
Superficial incisional infection	172 (0.44%)	152 (0.43%)	20 (0.56%)	0.23
Deep incisional infection	40 (0.1%)	38 (0.11%)	2 (0.06%)	0.58
Organ/space infection	25 (0.06%)	24 (0.07%)	1 (0.03%)	0.72
Wound disruption	10 (0.03%)	10 (0.03%)	0 (0%)	0.32
Thyroid-specific complications				
Post-op hypocalcemia	245 (0.62%)	235 (0.66%)	10 (0.28%)	**0.007**
Temporary RLN paresis	6 (0.02%)	5 (0.01%)	1 (0.03%)	0.43
General complications				
Sepsis/septic shock	112 (0.28%)	106 (0.3%)	6 (0.17%)	0.25
Pulmonary embolism	33 (0.08%)	30 (0.08%)	3 (0.08%)	0.99
Deep venous thrombosis	42 (0.11%)	38 (0.11%)	4 (0.11%)	0.79
Pneumonia	111 (0.28%)	111 (0.31%)	0 (0%)	**<0.001**
Unplanned intubations	172 (0.44%)	165 (0.46%)	7 (0.2%)	**0.028**
Ventilator > 48 h	94 (0.24%)	88 (0.25%)	6 (0.17%)	0.47
Urinary tract infection	105 (0.27%)	95 (0.27%)	10 (0.28%)	0.86
Acute renal failure	7 (0.02%)	6 (0.02%)	1 (0.03%)	0.49
Progressive Renal Insufficiency	11 (0.03%)	11 (0.03%)	0 (0%)	0.61
Cardiac arrest requiring CPR	24 (0.06%)	21 (0.06%)	3 (0.08%)	0.47
Myocardial Infarction	19 (0.05%)	18 (0.05%)	1 (0.03%)	0.56
CVA/stroke	17 (0.04%)	17 (0.05%)	0 (0%)	0.39
**Hospital admission**				
Outpatient procedure	23,860 (60.69%)	21,398 (59.85%)	2462 (69.14%)	**<0.001**
Length of stay				
1 day	32,861 (83.6%)	29,613 (82.8%)	3248 (91.2%)	**<0.001**
>1 day	6453 (10.4%)	313 (8.8%)	5140 (17.2%)	
Still in hospital > 30 days	53 (0.13%)	48 (0.13%)	5 (0.14%)	0.81
**Post-discharge**				
Reoperation	433 (1.1%)	410 (1.1%)	23 (0.6%)	**0.007**
Unplanned reoperation	361 (0.92%)	342 (0.96%)	19 (0.53%)	**0.012**
Readmission	992 (2.5%)	935 (2.6%)	57 (1.6%)	**<0.001**
Unplanned readmission	641 (1.6%)	609 (1.7%)	32 (0.9%)	**<0.001**
**Survival outcomes**				
30-day mortality	39 (0.1%)	36 (0.1%)	3 (0.08%)	0.77

Data are presented as number (percentage), mean and standard deviation (SD), or median and interquartile range (IQR). Two-sided Chi-square, Student’s t, and Mann–Whitney U tests were used. Bold *p*-values indicate statistical significance < 0.05. TT: total thyroidectomy; cT: completion thyroidectomy; AI/AN: American Indian or Alaska Native; COPD: chronic obstructive pulmonary disease; CVA: cerebrovascular accidents; ASA: American Society of Anesthesiologists classification; RLN: recurrent laryngeal nerve; DVT/PE: deep vein thrombosis and pulmonary embolism; CPR: cardiopulmonary resuscitation; CVA: cerebrovascular accidents; OR: operation room; NA: not applicable/unknown.

**Table 3:** Postoperative hypocalcemia risk stratified by type of neck dissection among total thyroidectomy groups.

Group	Total Count	Hypocalcemia		*p*-Value	*Relative Risk*	*p*-Value
		Negative	Positive		RR (95%CI)	
**Comparison 1**						
Completion thyroidectomy	3561	3551 (9.7)	10 (0.3)	--	*Reference*	--
TT without neck dissection	21,335	21,199 (99.4)	136 (0.6)	**0.009**	2.27 (1.19–4.31)	**0.012**
TT with neck dissection	14,418	14,319 (99.3)	99 (0.7)	**0.005**	2.44 (1.27–4.68)	**0.007**
TT with central lymphadenectomy	12,548	12,459 (99.3)	89 (0.7)	**0.003**	2.52 (1.31–4.85)	**0.005**
TT with lateral lymphadenectomy	1870	1860 (9.5)	10 (0.5)	0.14	1.90 (0.79–4.56)	0.15
**Comparison 2**						
TT without neck dissection	21,335	21,199 (99.4)	136 (0.6)	--	*Reference*	--
TT with neck dissection	14,418	14,319 (99.3)	99 (0.7)	0.61	1.07 (0.83–1.39)	0.57
**Comparison 3**						
TT with central lymphadenectomy	12,548	12,459 (99.3)	89 (0.7)	--	*Reference*	--
TT with lateral lymphadenectomy	1870	1860 (9.5)	10 (0.5)	0.48	0.75 (0.39–1.44)	0.39

Binary pairwise comparisons were performed using Chi-square test. Relative risk (RR), its standard error, and its 95% confidence interval were computed. Bold *p*-values indicate statistical significance < 0.05.

**Table 4:** Risk of short-term and long-term postoperative complications in TriNetX dataset.

Complications	TT	cT	*p*-Value	RR	95%CI	*p*-Value
**Overall**				**TT versus cT**		
Transient hypocalcemia	10,636 (34.4%)	999 (20.1%)	**<0.001**	1.72	1.62–1.81	**<0.001**
Permanent hypocalcemia	4205 (13.6%)	541 (10.9%)	**<0.001**	1.25	1.15–1.36	**<0.001**
Transient RLN injury	1603 (5.2%)	279 (5.6%)	0.12	0.93	0.82–1.05	0.22
Permanent RLN injury	759 (2.5%)	134 (2.7%)	0.16	0.91	0.76–1.09	0.32
**Non-obese**						
Transient hypocalcemia	8560 (35.2%)	807 (20.9%)	**<0.001**	1.68	1.57–1.79	**<0.001**
Permanent hypocalcemia	1478 (6.1%)	190 (4.9%)	**0.004**	1.23	1.06–1.43	**0.005**
Transient RLN injury	1218 (5%)	199 (5.2%)	0.34	0.97	0.84–1.12	0.67
Permanent RLN injury	2677 (11%)	375 (9.7%)	**0.017**	1.13	1.02–1.25	**0.018**
**Obese**						
Transient hypocalcemia	2076 (31.7%)	192 (17.1%)	**<0.001**	1.85	1.62–2.11	**<0.001**
Permanent hypocalcemia	1098 (16.8%)	127 (11.3%)	**<0.001**	1.48	1.25–1.76	**<0.001**
Transient RLN injury	385 (5.9%)	80 (7.1%)	0.06	0.82	0.65–1.04	0.10
Permanent RLN injury	173 (2.6%)	37 (3.3%)	0.11	0.80	0.56–1.14	0.21

Data are presented as counts (percentages) of total thyroidectomy (TT) and completion thyroidectomy (cT) patients. Two-sided Chi-square test was used for comparison. Bold *p*-values indicate statistical significance < 0.05. Relative risk (RR) and 95% confidence interval (CI) for risk ratio of TT versus cT. Hypocalcemia was defined as calcium < 8.5 mg/dL. Duration was defined as transient (lasting < 6 months) versus permanent (>6 months). RLN: recurrent laryngeal nerve. These complications were reported in 30,897 TT (24,343 non-obese and 6554 obese) and 4976 cT patients (3854 non-obese and 1122 obese).

**Table 5:** Risk of postoperative complications stratified by extent of neck dissection in TriNetX dataset.

Complications	Group 1	Group 2	*p*-Value	RR	95%CI	*p*-Value
	**TT with ND**	**cT**		**TT with ND versus cT**		
Transient hypocalcemia	5027 (39.6%)	999 (20.1%)	**0.002**	1.97	1.86–2.09	**<0.001**
Permanent hypocalcemia	1956 (15.49%)	541 (10.9%)	**0.032**	1.42	1.30–1.55	**<0.001**
Transient RLN injury	913 (7.2%)	279 (5.6%)	**<0.001**	1.28	1.13–1.46	**0.002**
Permanent RLN injury	427 (3.4%)	134 (2.7%)	**<0.001**	1.24	1.03–1.51	**0.022**
	**Limited ND**	**Radical ND**		**Limited versus radical ND**		
Transient hypocalcemia	4422 (38.3%)	605 (53.7%)	**<0.001**	1.41	1.32–1.49	**<0.001**
Permanent hypocalcemia	1761 (15.2%)	195 (17.3%)	0.06	1.14	0.99–1.3	0.06
Transient RLN injury	786 (6.8%)	127 (11.3%)	**<0.001**	1.66	1.39–1.98	**<0.001**
Permanent RLN injury	359 (3.1%)	68 (6%)	**<0.001**	1.95	1.51–2.5	**<0.001**

Data are presented as counts (percentages) comparing (a) total thyroidectomy (TT) with neck dissection (ND) and completion thyroidectomy (cT) patients or (b) thyroidectomy with limited and radical neck dissection. Two-sided Chi-square test was used for comparison. Relative risk (RR) and 95% confidence interval (CI). Hypocalcemia was defined as calcium < 8.5 mg/dL. Duration was defined as transient (lasting < 6 months) versus permanent (>6 months). RLN: recurrent laryngeal nerve. Bold *p*-values indicate statistical significance < 0.05.

**Table 6:** Impact of timing of completion thyroidectomy on complication rates.

Complications	Timing of cT	Count	Complication Rate	RR (95%CI)	*p*-Value
**Transient hypocalcemia**	1–15 days	48	18 (37.5%)	1.5 (0.95–2.32)	0.08
	16–30 days	41	13 (31.7%)	1.25 (0.75–2.1)	0.39
	1–3 months	150	32 (21.3%)	0.84 (0.56–1.26)	0.41
	3–6 months	100	27 (27%)	1.07 (0.71–1.61)	0.76
	>6 months	170	43 (25.3%)	*Reference*	
**Permanent hypocalcemia**	1–15 days	48	17 (35.4%)	1.15 (0.72–1.73)	0.48
	16–30 days	41	11 (26.8%)	0.84 (0.49–1.47)	0.55
	1–3 months	150	27 (18%)	0.57 (0.38–0.85)	**0.006**
	3–6 months	100	15 (15%)	0.47 (0.28–0.79)	**0.004**
	>6 months	170	54 (31.8%)	*Reference*	
**Transient RLN injury**	1–15 days	48	9 (18.8%)	1.52 (0.74–3.1)	0.25
	16–30 days	41	4 (9.8%)	0.79 (0.29–2.18)	0.46
	1–3 months	150	12 (8%)	0.65 (0.33–1.27)	0.21
	3–6 months	100	6 (6%)	0.49 (0.2–1.16)	0.11
	>6 months	170	21 (12.4%)	*Reference*	
**Permanent RLN injury**	1–15 days	48	3 (6.3%)	0.41 (0.13–1.29)	0.13
	16–30 days	41	3 (7.3%)	0.48 (0.15–1.5)	0.21
	1–3 months	150	9 (6%)	0.39 (0.19–0.81)	**0.012**
	3–6 months	100	5 (5%)	0.33 (0.13–0.82)	**0.018**
	>6 months	170	26 (15.3%)	*Reference*	

Data presented as number of patients (percentage with complication). Relative risk and 95% CI compared to reference group of delayed completion thyroidectomy at >6 months. Bold *p*-values indicate statistical significance < 0.05.

## Data Availability

Data are available upon appropriate request.

## Supplementary Materials

The following supporting information can be downloaded at https://www.mdpi.com/article/10.3390/cancers16122250/s1: Figure S1: Predictors for hypocalcemia; Figure S2: Predicting factors for mortality; Figure S3: Comparing complication rates with early (<3 months) versus delayed (>3 months) completion thyroidectomy. Table S1. The CPT codes for thyroidectomy in NSQIP and TriNetX databases; Table S2. Independent risk factors for developing postoperative complications; Supplementary Table S3. Subgroup analysis for patients with small tumor size ≤ 2 cm; Supplementary Table S4. Multivariate logistic regression for postoperative hypocalcemia; Table S5. Baseline characteristics of patients in TriNetX database.
