# Supplementary material for: Initial versus Staged Thyroidectomy for Differentiated Thyroid Cancer: A Retrospective Multi-Dimensional Cohort Analysis of Effectiveness and Safety
Source: Cancers (Basel). 2024 Jun 18;16(12):2250. doi: 10.3390/cancers16122250 (PMC11201776; doi:10.3390/cancers16122250)
Supplement: Supplementary file 1 [file cancers-16-02250-s001.zip › cancers-3026001-supplementary.pdf]

Supplementary Table S1. The CPT codes for thyroidectomy in NSQIP and TriNetX databases.

CPT Code	Description	NSQIP	TriNetX
60240, 60270, 60271	Total thyroidectomy	21,335	19,021
60252	Total thyroidectomy with limited neck dissection	12,548	11,560
60254	Total thyroidectomy with radical neck dissection	1,870	1,126
60260	Completion thyroidectomy	3,561	5,039
**Total**		**39,314**	**36,746**

Supplementary Table S2. Independent risk factors for developing postoperative complications.

Risk factor	OR	LL	UL	*p*-value
Age, years	0.98	0.94	1.02	0.28
Male vs female	0.41	0.05	3.62	0.42
Black vs White	1.97	0.63	6.22	0.25
Body mass index, Kg/m^2	1.00	0.93	1.07	0.95
Hashimoto disease	0.88	0.20	4.01	0.87
Maximum diameter	0.78	0.46	1.32	0.36
Nodal infiltration vs none	1.07	0.12	9.76	0.95
Multifocal vs unifocal lesion	2.11	0.66	6.73	0.21

Logistic regression analysis was performed, and data was reported as odds ratio (OR) and 95% confidence interval. LL: lower limit; UL: upper limit of the 95% confidence interval. *P*-value was set significant <0.05.

Supplementary Table S3. Subgroup analysis for patients with small tumor size ≤2 cm.

Characteristics	Total (N=93)	TT (N=40)	CT (N=53)	*p*-value
**Complication rate**				
No complications	80 (86)	31 (77.5)	49 (92.5)	0.07
Complications	13 (14)	9 (22.5)	4 (7.5)	
**Type of complication**				
Temporary RLN dysfunction	10 (10.8)	6 (15)	4 (7.5)	0.32
Temporary hypocalcemia	5 (5.4)	5 (12.5)	0 (0.0)	**0.013**

Data is reported as count (percentage). Two-sided Chi-square was used. TT: total thyroidectomy; cT: completion thyroidectomy; RLN: recurrent laryngeal nerve injury.

Supplementary Table S4. Multivariate logistic regression for postoperative hypocalcemia.

Predictor risk factors	OR	LL	UL	*p*-value
Age ≥ 55 vs <55 years	0.60	0.45	0.79	**<0.001**
Male vs female	0.41	0.28	0.61	**<0.001**
Obesity	1.96	1.52	2.53	**<0.001**
Completion vs total thyroidectomy	0.42	0.22	0.79	**0.007**

Bold *p*-values indicate statistical significance <0.05.

Supplementary Table S5. Baseline characteristics of patients of TriNetX database.

Characteristics	TT	cT	*p*-value
**Number**	31,707	5,039	
**Demographics**			
Age, years	56 ± 17	57 ± 16	**<0.001**

<30	2,020 (6.36%)	281 (5.58%)	**<0.001**
30-49	9,162 (28.84%)	1,438 (28.54%)	
50-69	13,599 (42.8%)	2,077 (41.22%)	
≥70	6,926 (21.8%)	1,243 (24.67%)	
Sex			
Male	8,561 (27%)	1,411 (28%)	**<0.001**
Female	22,828 (72%)	3,578 (71%)	
Unknown	318 (1%)	49 (1%)	
Race			**<0.001**
White	23,146 (73%)	3,628 (72%)	
Black	1,902 (6%)	454 (9%)	
Asian	1,585 (5%)	252 (5%)	
AI/AN	317 (1%)	50 (1%)	
Other race	1,268 (4%)	151 (3%)	
Unknown	3,488 (11%)	504 (10%)	
Ethnicity			
Hispanic/Latino	3,488 (11%)	454 (9%)	**<0.001**
Not Hispanic/Latino	20,927 (66%)	3527 (70%)	
Unknown	7,293 (23%)	1,058 (21%)	
BMI, Kg/m^2	30.1 ± 7.1	30.7 ± 7	0.30
**Comorbidities**			
Obesity (BMI >30Kg/m^2)	6,544 (20.6%)	1,122 (22.3%)	**0.004**
Diabetes	6,761 (21%)	1,064 (21%)	0.36
Hypertension requiring medication	14,889 (47%)	2,401 (48%)	**<0.001**
Cerebrovascular accidents	2,793 (9%)	431 (9%)	0.27
Chronic lower respiratory disease	7,530 (24%)	1,193 (24%)	0.45
Chronic kidney disease	2,648 (8%)	412 (8%)	0.34
**Pathology**			
Positive lymph node metastasis	703/5,025 (13.8%)	133/747 (17.8%)	**0.002**
Positive distal metastasis	112/3,373 (3.3%)	12/590 (2.0%)	**0.048**

Data is presented as number (percentage) or mean and standard deviation (SD). Two-sided Chi-Square and Student's t tests were used. Bold *p*-values indicate statistical significance <0.05. TT: total thyroidectomy, cT: completion thyroidectomy, AI/AN: American Indian or Alaska Native, NA: not applicable/unknown.

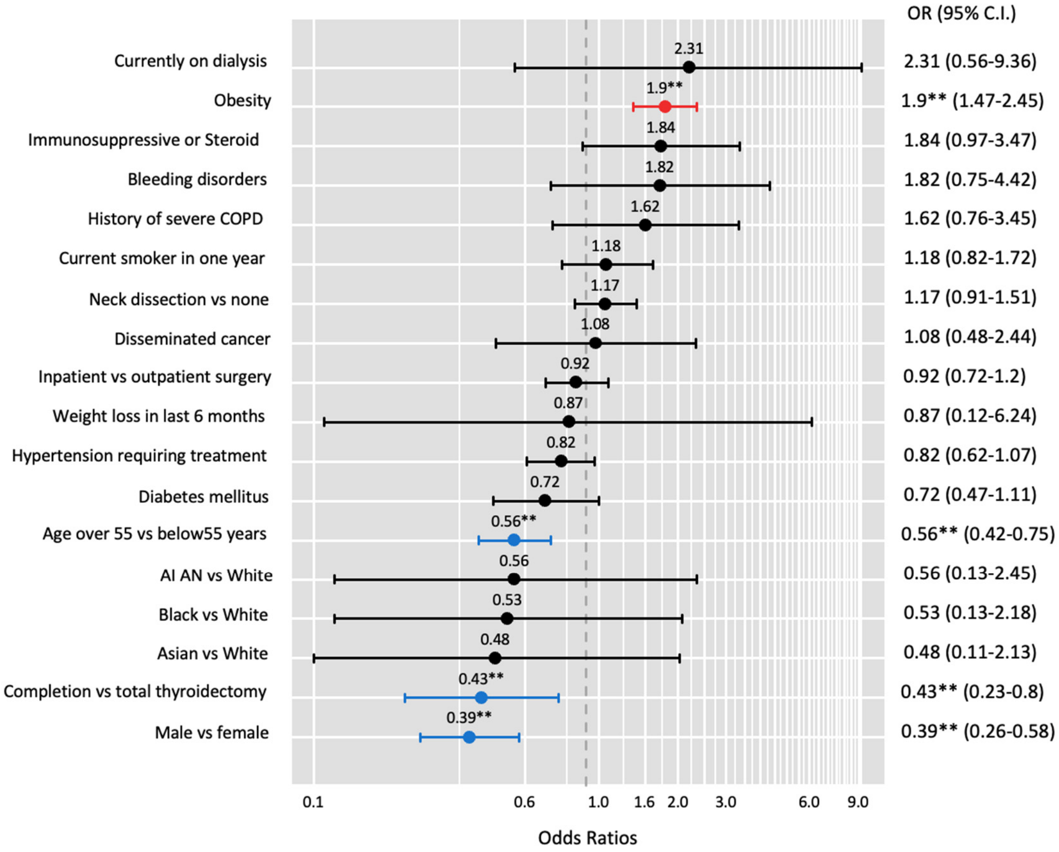

Supplementary Fig. S1. Predictors for hypocalcemia. Univariate logistic regression was performed, and data was reported as odds ratio (OR) and 95% confidence interval (C.I). AI/AN: American Indian or Alaska Native, COPD: chronic obstructive pulmonary disease. *p<0.05; **p<0.001.

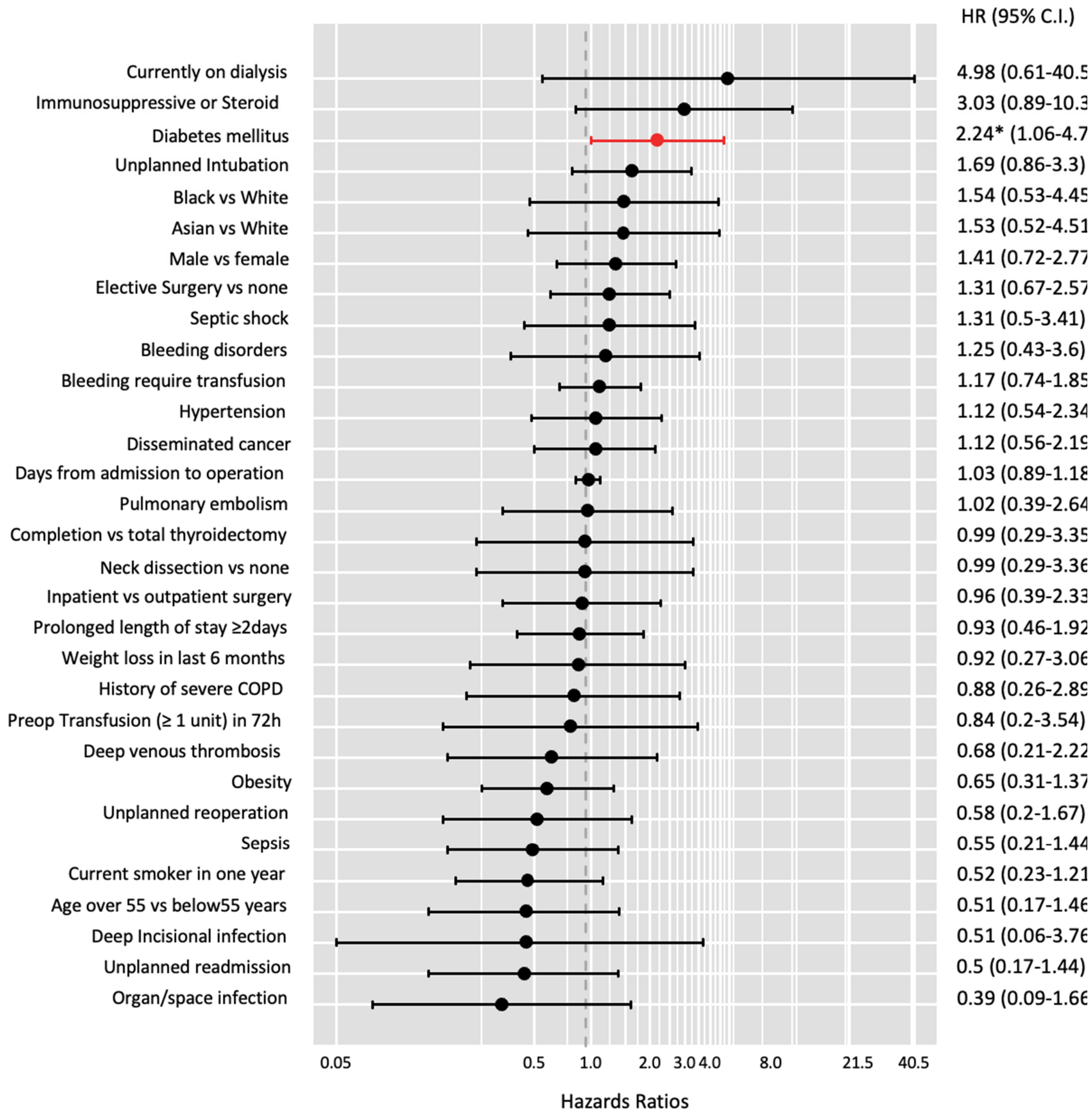

Supplementary Fig. S2. Predicting factors for mortality. Cox Proportionate Regression analysis was performed. COPD: chronic obstructive pulmonary disease, HR: Hazards ratio, LL: lower limit, UL: upper limit of 95% confidence interval.

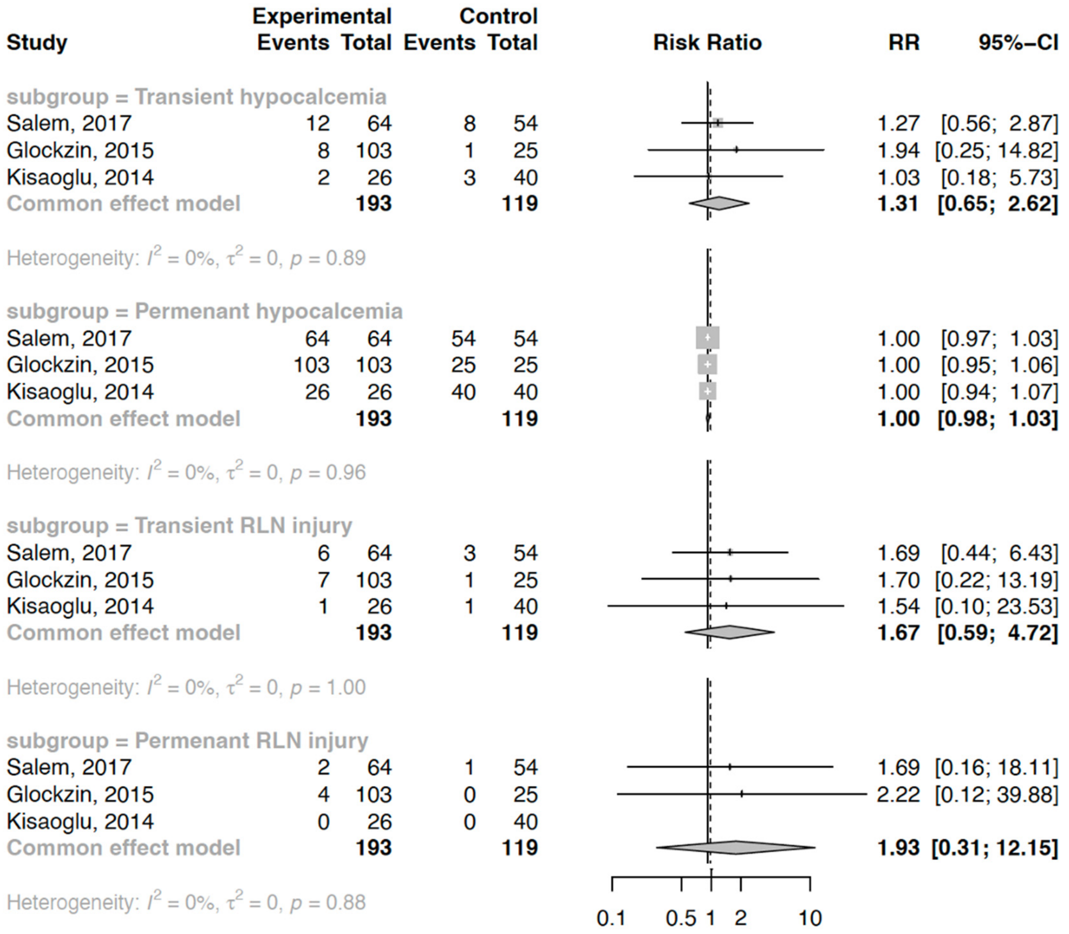

Supplementary Fig. S3. Comparing complication rates with early (<3 months) versus delayed (>3 months) completion thyroidectomy. Pairwise comparison of relative risk (RR) with 95% confidence interval (CI) for postoperative complications including transient and permanent hypocalcemia and recurrent laryngeal nerve (RLN) injury. Early completion thyroidectomy was defined as <3 months between initial surgery and completion, while delayed was defined as >3 months. RRs compare the risk of each complication with early versus delayed completion thyroidectomy timing. Significance denoted if 95% CI does not cross 1.
